# Induction of Long-Term Protective Immune Responses by Influenza H5N1 Virus-Like Particles

**DOI:** 10.1371/journal.pone.0004667

**Published:** 2009-03-02

**Authors:** Sang-Moo Kang, Dae-Goon Yoo, Aleksandr S. Lipatov, Jae-Min Song, C. Todd Davis, Fu-Shi Quan, Li-Mei Chen, Ruben O. Donis, Richard W. Compans

**Affiliations:** 1 Department of Microbiology and Immunology and Emory Vaccine Center, Emory University School of Medicine, Atlanta, Georgia, United States of America; 2 Influenza Division, National Center for Immunization and Respiratory Diseases, Centers for Disease Control and Prevention, Atlanta, Georgia, United States of America; Karolinska Institutet, Institution for Laboratory Medicine, Sweden

## Abstract

**Background:**

Recurrent outbreaks of highly pathogenic H5N1 avian influenza virus pose a threat of eventually causing a pandemic. Early vaccination of the population would be the single most effective measure for the control of an emerging influenza pandemic.

**Methodology/Principal Findings:**

Influenza virus-like particles (VLPs) produced in insect cell-culture substrates do not depend on the availability of fertile eggs for vaccine manufacturing. We produced VLPs containing influenza A/Viet Nam1203/04 (H5N1) hemagglutinin, neuraminidase, and matrix proteins, and investigated their preclinical immunogenicity and protective efficacy. Mice immunized intranasally with H5N1 VLPs developed high levels of H5N1 specific antibodies and were 100% protected against a high dose of homologous H5N1 virus infection at 30 weeks after immunization. Protection is likely to be correlated with humoral and cellular immunologic memory at systemic and mucosal sites as evidenced by rapid anamnestic responses to re-stimulation with viral antigen in vivo and *in vitro*.

**Conclusions/Significance:**

These results provide support for clinical evaluation of H5N1 VLP vaccination as a public health intervention to mitigate a possible pandemic of H5N1 influenza.

## Introduction

Influenza A is one of the major threats to human and animal health due to its high transmissibility, and the potentially severe morbidity and mortality among susceptible hosts. Early vaccination is one of the most effective means to mitigate a future influenza pandemic [1]. Licensed influenza vaccines are chemically inactivated detergent-solubilized virions composed of hemagglutinin (HA) and lesser amounts of other virion proteins (i.e. neuraminidase (NA)). Another vaccine licensed only for seasonal influenza is a live, attenuated influenza virus vaccine (FluMist®) intended for intranasal administration [2], [3]. Both types of licensed influenza vaccines rely on fertilized chicken eggs as substrates for production [4]. Licensed seasonal influenza vaccines provide incomplete protection [5], probably due in part to the limited mucosal immunity and cytotoxic T cell immunity. Furthermore, the protective immunity elicited by inactivated vaccines is of short duration [4], [6].

Wild birds are a source for 16 influenza A HA subtypes, representing a large reservoir for novel glycoproteins to which the human immune system is naïve [7]–[9]. In 1997, human infections with avian influenza A H5N1 viruses resulted in 6 fatal deaths out of 18 confirmed cases [10], [11]. Since 2003, there have been 243 deaths out of 385 human cases of avian influenza A H5N1 virus, a case fatality ratio higher than that of many viral hemorrhagic fevers [12]. Outbreaks of highly pathogenic avian influenza viruses in poultry and confirmed human cases are occurring at unprecedented rates, and increase the risk of pandemic emergence if the H5N1 virus acquires the capability to spread from person to person [13], [14]. Therefore, an effective vaccine against H5N1 influenza viruses would meet an important public health need.

Development and manufacturing of effective H5N1 vaccines pose significant practical challenges. Handling of highly pathogenic H5N1 influenza viruses requires biosafety level 3 laboratory facilities. Manufacturing capacity is likely to be limited by insufficient availability of fertile eggs to meet the surge in vaccine demand during a pandemic. Unadjuvanted inactivated H5N1 vaccines require six times higher antigen doses than conventional seasonal vaccines to elicit a comparable immune response; the use of proprietary adjuvants may reverse this situation if they are approved by regulatory authorities [15]–[21]. In attempts to overcome these obstacles, several strategies have been explored including the use of a low pathogenic avian influenza virus [22], baculovirus- expressed H5 HA [23], recombinant adenoviruses [24], [25] and the production of attenuated live seed virus with modified H5 HA [17], [26], [27].

As an alternative to conventional egg-based influenza vaccine manufacturing, our laboratory and others have developed non-replicating virus-like particles (VLPs). VLPs are safer than virion-derived vaccines by virtue of lacking a viral genome and retain high immunogenicity because the HA antigen is presented to the host in a native particulate form without chemical inactivation. The expression of influenza proteins in a recombinant baculovirus system yields VLPs which present conformational epitopes of surface proteins to the immune system comparable to those of live virions. Recent studies demonstrated that VLPs containing influenza glycoproteins (HA or both HA and NA) and the influenza matrix protein M1 induced high titers of virus-specific antibodies in vaccinated mice or ferrets and provided immunized animals with protection against otherwise lethal experimental infections [28]–[32].

To examine the feasibility of a VLP vaccine against a potentially pandemic influenza virus, we generated influenza VLPs containing A/Viet Nam/1203/04 hemagglutinin (HA) and neuraminidase (NA) as well as M1, and investigated their immunogenicity, long-term protective immunity, and immunologic memory responses. Intranasal delivery of two doses of H5N1 VLPs to mice induced long-lived antibody responses and provided protection against lethal challenge infections with wild type influenza H5N1 virus in dose-range experiments. The H5N1 VLPs were observed to induce humoral and cellular memory responses, which were rapidly recalled upon re-exposure to viral antigen *in vivo*.

## Results

### Production of H5N1 VLPs

**Figure 1:**
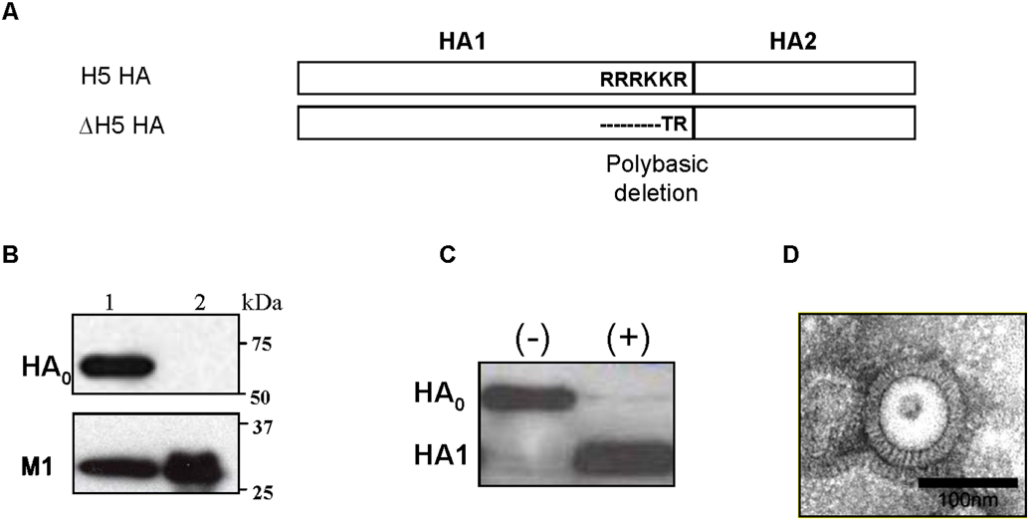
Characterization of influenza H5N1 VLPs. A) A schematic diagram of wild type and mutant H5 HA. The mutant H5 HA is showing a deletion of polybasic amino acids (RRRKK) in the cleavage region of HA (ΔH5 HA). B) Western blot analysis of purified H5N1 VLPs. Lane 1, Influenza H5N1 VLPs. Lane 2, M1 VLPs lacking H5 HA. C) Cleavage of HA in VLPs. H5N1 VLPs were incubated without (−) or with (+) TPCK-treated trypsin (2.0 µg/ml trypsin), resolved on SDS-PAGE, and probed by western blot. D) Negative stain electron microscopy of influenza H5N1 VLPs.

Influenza H5N1 VLPs were produced in insect cells co-infected with three rBVs, each expressing A/Viet Nam/1203/2004, subtype H5N1 (VN/04) virus HA, NA, or M1. Purified H5N1 VLPs were found to contain HA (Fig. 1B) and NA (data not shown) proteins as well as M1, as indicated by the SDS-PAGE and western blot analysis. The H5 HA lacking a polybasic cleavage site in VLPs was expressed as a single polypeptide (HA_0_) that was readily cleaved into HA subunits upon trypsin treatment (Fig. 1C). H5N1 VLP preparations hemagglutinated chicken erythrocytes with titers ∼128 to 256 in samples containing 1 µg of VLPs. In addition, negatively stained preparations of H5N1 VLPs examined by transmission electron microscopy revealed particles of 80–120 nm in diameter with spikes on the surface resembling influenza virions (Fig. 1D). Therefore, influenza H5N1 VLPs produced by cells infected with rBVs were similar to influenza virions in structure, size, and morphology despite lacking the viral ribonucleoprotein components.

### Antibody responses to influenza H5N1 VLPs

**Figure 2:**
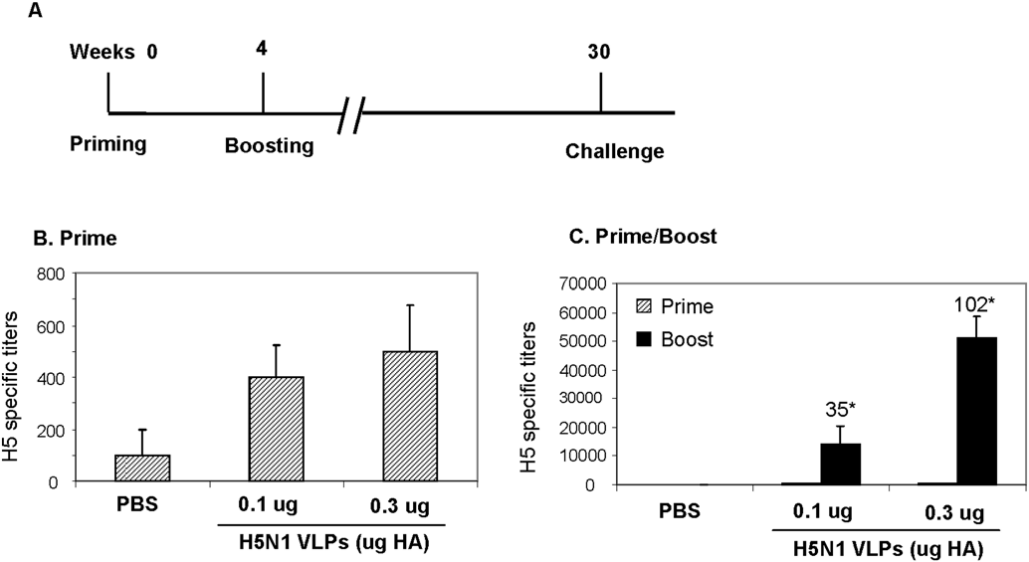
H5N1 Influenza virus specific total serum IgG antibody responses. A) Timeline for immunization and challenge. B) Influenza H5N1 virus specific serum IgG antibody titers at two weeks after priming by ELISA. Groups of mice (n = 6) were intranasally immunized with H5N1 VLPs (0.1 µg and 0.3 µg of HA as indicated). PBS is a mock control group of mice mock-immunized with buffer only. C) Serum IgG antibody titers to inactivated rgΔH5N1 influenza virus by ELISA at 2 weeks after boost immunization. Error bars indicate standard deviation. 0.1 µg HA and 0.3 µg HA indicate groups of mice immunized with H5N1 VLPs containing 0.1 µg and 0.3 µg of H5 HA respectively. * Fold increases in virus-specific antibody titers as compared to levels after priming.

Mice were immunized intranasally with H5N1 VLPs containing 0.1 µg or 0.3 µg H5 HA and a boost was given 4 weeks after the priming dose. All mice remained healthy and showed no signs of abnormal behavior after vaccination with pandemic influenza H5N1 VLPs. The first (priming) immunization of mice with H5N1 VLPs induced low but detectable levels of antibodies as measured by ELISA plates coated with inactivated rgΔH5N1 virus antigen (Materials and methods, Fig. 2A, 2B). The levels of virus-specific antibodies were greatly increased two weeks after boost immunization (Fig. 2C); the 0.3 µg HA group showed significant increases in excess of 100 fold in ELISA titers compared to those observed after priming whereas the 0.1 µg HA group showed increases of 35 fold in antibody titers. Therefore, these results indicate that an intranasal prime– boost immunization regimen with VLPs can induce robust humoral antibody responses against H5N1 virus.

**Figure 3:**
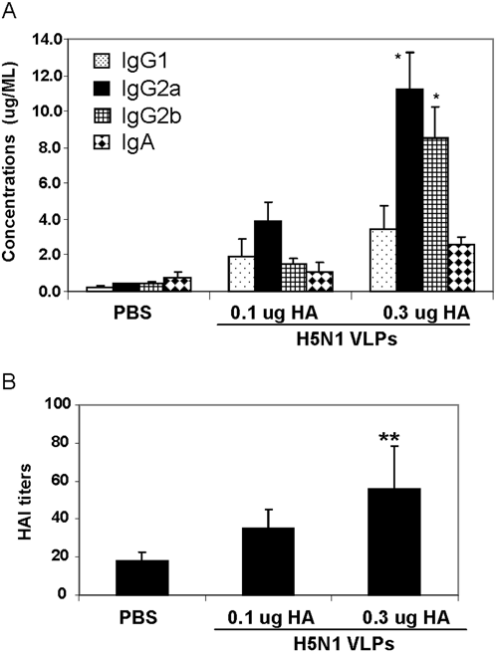
Isotypes of antibody responses induced by H5N1 VLP immunization. Serum samples collected at week 28 post immunization were used to determine levels of isotype antibodies specific to influenza H5N1 virus (A, n = 6) and hemagglutination inhibition titers (B, n = 5). Error bars are shown to indicate standard deviation. * (A) Indicates statistically significant differences between 0.1 µg HA and 0.3 µg HA groups (p<0.02). ** (B) statistical significance between 0.3 µg HA and PBS mock groups (p<0.02).

Dissection of the influenza-specific IgG isotypes revealed that at 28 weeks after immunization IgG2a and IgG2b are the dominant antibody components elicited by the influenza H5N1 VLPs, in agreement with previous reports [31], [33]. Interestingly, increased IgG2a and IgG2b are responsible for the enhanced antibody levels in the 0.3 µg HA group as compared to those in the 0.1 µg HA group (Fig. 3A).

The functional significance of the antibody responses induced by intranasal immunization with VLPs was evaluated by hemagglutination inhibition (HAI) and neutralization assays. Moderate HAI titers with a mean of 56 and standard error of 21 were elicited at 2 weeks after boost in the 0.3 µg group. Lower HAI titers (35+/−10) were detected in the 0.1 µg group, although statistically not significant compared to those in naïve control (Fig. 3B). In the microneutralization assay, a low titer of 40 against the homologous VN/04 virus was elicited by the 0.3 µg dose vaccination. These results demonstrated that H5N1 VLPs were immunogenic when given intranasally to mice without adjuvant and could induce moderate to low levels of HAI titers which might have a correlation with host protection.

### Long-lived protective immunity induced by influenza H5N1 VLPs

**Figure 4:**
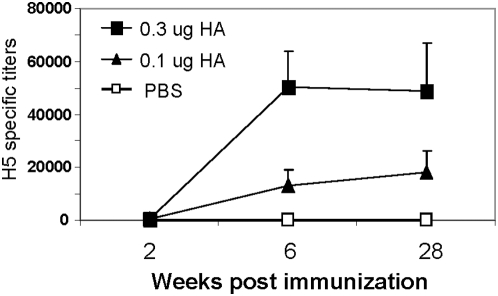
Long-term maintenance of IgG antibodies specific to influenza H5N1 virus. Serum samples collected at week 2 post priming (week 2), at week 2 post boosting (week 6), and at week 28 before challenge infection were used to determine antibody levels specific to H5N1 virus by ELISA with whole H5N1 inactivated virus antigen. Antibody titers are shown as described in Materials and methods, and error bars indicate standard deviation (n = 6).

A goal for vaccination is to induce long-lived protective immunity. To evaluate the longevity of the response to intranasal VLP vaccine, prime-boost immunized mice kept in isolation were bled after 7 months to determine the duration of H5N1 virus specific antibody levels and to compare with titers at 2 weeks after boosting. The levels of influenza virus-specific antibodies remained virtually unchanged at 28 weeks after immunization (Fig. 4).

**Figure 5:**
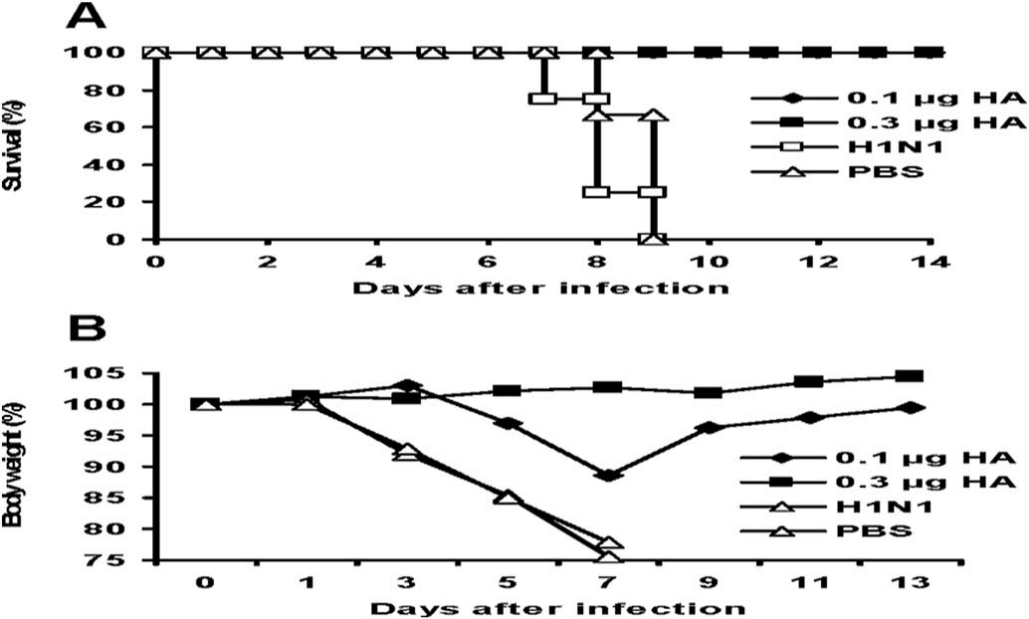
Protection of immunized mice from lethal infection with VN/04 influenza virus. At week 30 post immunization, H5N1 VLP immunized and control (PBS or 5 µg inactivated PR/8/34 H1N1 virus) groups of mice were intranasally infected with 100 MLD_50_ of wild type H5N1 influenza virus. Mice were monitored daily for 14 days to determine the survival rate (A) and body weight changes (B). The percent of body weight loss calculated from the initial body weight taken at day 0. Data are the average from 6 individual mice. 0.1 µg HA and 0.3 µg HA indicate the groups of mice immunized with H5N1 VLPs containing 0.1 µg and 0.3 µg of H5 HA respectively.

To determine whether intranasal H5N1 VLP vaccination could elicit protection against lethal infection, immunized mice were challenged with a highly lethal dose (100 50% mouse lethal doses [MLD_50_]) of the wild type VN/04 virus at 30 weeks after the boost immunization (Fig. 5). The control animals that received PBS or inactivated PR8/34 H1N1 virus showed clinical signs of severe disease and significant body weight loss starting on day 3 after virus inoculation, and died or reached the humane euthanasia endpoint 7–9 days after challenge. In contrast, all mice immunized with the low dose of H5N1 VLPs (0.1 µg HA) remained alive by 14 days after challenge; only a transient body weight loss was noted at day 7 but the animals recovered completely during the following week. Mice vaccinated with the 0.3 µg dose of H5N1 VLPs were completely protected; no weight loss or apparent illness symptoms were noted. Taken together, these results provide evidence that H5N1 VLPs are an effective immunogen to induce long-term protective immunity against a highly pathogenic H5N1 avian influenza virus in mice.

### Replication of VN/04 virus in the lungs and systemic sites of immunized mice

**Figure 6:**
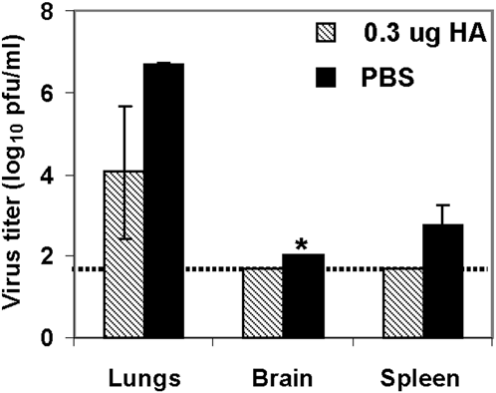
Replication of wild type VN/04 virus in mice immunized with H5N1 VLPs. Mice intranasally immunized twice with influenza H5N1 VLPs containing 0.3 µg H5 HA were infected with 100 MLD_50_ dose of VN/04 virus at 4 weeks after the second immunization. At day 4 after virus inoculation, mice were euthanized and lung, brain, and spleen were collected from individual mice. Virus titer in the lung homogenate was determined by plaque assay on MDCK cells. Data are the mean virus titers from 4 individual mice+/−Standard Error of the Mean (SEM). Dashed horizontal line indicated lower limit of detection. *, Indicated virus was isolated from one mouse.

Previous studies demonstrate that VN/04 H5N1 virus is highly pathogenic for mice as a result of its rapid systemic spread [34], [35]. We investigated whether intranasal immunization with H5N1 VLPs could prevent local replication of VN/04 virus in the respiratory tract and its systemic spread to internal organs and brain tissue. Mice vaccinated twice with 0.3 µg H5N1 VLPs were challenged with 10^3^ pfu (100 MLD_50_) VN/04 virus and the animals were euthanized at day 4 after inoculation to collect lungs, brain and spleen to quantify the presence of wild type VN/04 virus. Viral titers in lungs were reduced by approximately 1000 fold in the H5N1 VLP immunized group compared to those in naïve mice (Fig. 6). Viral titers in brain and spleen of VLP-immunized mice were below the detection limit whereas significant viral titers were in spleens of control (PBS-inoculated) animals. Thus, our results show that immunization with H5N1 VLPs decreased viral titers in the lungs and prevented systemic spread of VN/04 H5N1 virus in mice.

### Induction of plasma and memory B cells

**Figure 7:**
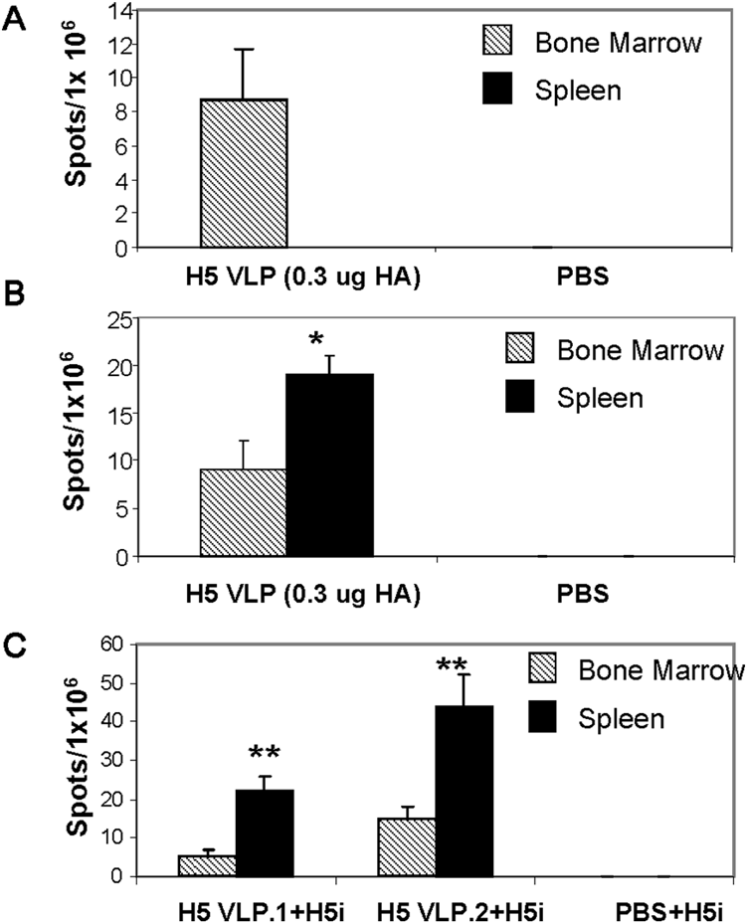
Generation of virus specific plasma and memory B cells in mice immunized with H5N1 VLPs. A) Generation of virus specific antibody secreting plasma cells. Groups of mice (n = 6) were intranasally immunized with influenza H5N1 VLPs (containing 0.3 µg HA) or PBS buffer at weeks 0 and 4. At 4 weeks post immunization, bone marrow and spleen cells were prepared and *in vitro* cultured for 18 hrs on plates coated with inactivated rgΔH5N1 virus to detect the antibody secreting cell spots. B) Antibody secreting cells after 3-day culture stimulation. The bone marrow and spleen cells were from the same group of mice as in A except the 3-day *in vitro* culture. C) Antibody secreting cells after 18 hrs culture. Groups of mice (n = 6) were intranasally immunized once (H5 VLP.1+H5i) or twice (H5 VLP.2+H5i) (weeks 0 and 4) with influenza H5N1 VLPs (containing 0.3 µg HA) or PBS buffer (PBS+H5i) and were administered with inactivated rgΔH5N1 virus (2.5 µg) 6 days earlier before euthanasia. *, ** indicate statistically significant differences between bone marrow and spleen (p<0.05 and p<0.01 respectively) as determined by Student's 2-tailed t-test.

Differentiation and expansion of B cells as a consequence of antigen exposure culminates with a fraction of the germinal center B cells trafficking to the bone marrow where antigen-specific antibody secreting plasma cells reside [36]–[38]. Maintenance of virus-specific antibodies and protective immunity for over 7 months prompted us to quantify antibody secreting plasma cells and memory B cells that might have been generated in mice immunized with influenza H5N1 VLPs. Bone marrow and spleen from mice immunized with H5N1 VLPs containing 0.3 µg HA were collected at 4 weeks post prime-boost immunization. Significant numbers of virus-specific antibody secreting bone marrow cells were noted from H5N1 VLP-immunized mice but not from PBS control mice within 18 hr in culture, suggesting that pre-existing plasma cells induced by VLP vaccination were actively secreting antibodies to H5N1 virus antigen (Fig. 7A). After 3 days of culture, virus-specific antibody secreting cells in splenocyte cultures surpassed those in bone marrow (Fig. 7B), which may represent rapidly differentiated virus-specific memory B cells. To determine the recall proliferative responses of memory B cells *in vivo*, mice were either primed only or prime-boosted with H5N1 VLPs intranasally and then 4 weeks later stimulated by intranasal delivery of 5 µg of inactivated rgΔH5N1 virus. Bone marrow and spleen cells were collected 6 days after antigenic stimulation with inactivated rgΔH5N1 virus and analyzed for virus specific antibody secreting cells. Significant numbers of antibody secreting cells from both bone marrow and spleens were detected in 18-hr cultures (Fig. 7C). Detection of virus-specific antibody secreting cells in spleens of mice that received a single dose (H5 VLP.1) suggests the induction of memory B cells after priming. Prime-and-boost immunized mice (H5 VLP.2) showed higher numbers of virus-specific antibody secreting recalled B cells as compared to mice that were primed only. Overall, these results indicate that H5N1 VLPs can induce H5 virus specific plasma cells as well as memory B cells.

### Anamnestic mucosal immune responses

**Figure 8:**
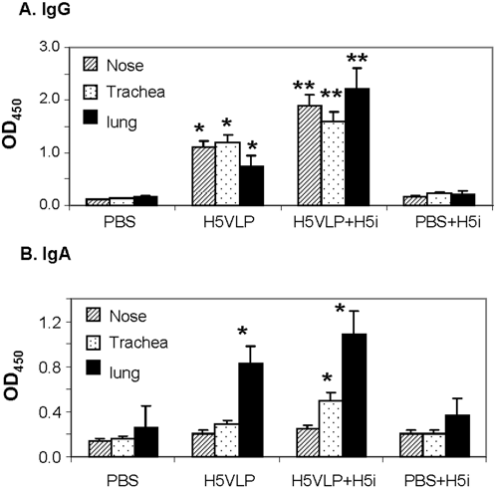
Mucosal antibody responses. A) Virus specific mucosal IgG. B) Virus specific mucosal IgA. H5VLP indicate a groups of mice (n = 6 for each group) that was intranasally immunized with H5N1 VLPs containing 0.3 µg H5 HA at weeks 0 and 4. PBS is a mock control. H5VLP+H5i and PBS+H5i indicate additional groups of mice that were intranasally administered with inactivated rgΔH5N1 virus (2.5 µg) 6 days earlier before euthanasia (in vivo viral antigen exposure). Nose, trachea, and lung samples from individual mouse were prepared in 200 µl, 200 µl, and 1000 µl PBS buffer respectively. Diluted samples (10×) were used to determine antibody responses on ELISA plates coated with inactivated rgΔH5N1 virus and optical densities were read at 450 nm. * p<0.05 compared to the PBS control. ** p<0.05 compared to H5VLP.

Mucosal immune responses are important for host protection from infection by blocking initial multiplication of the pathogen at the portal of entry. We analyzed mucosal respiratory secretions from a group of mice that was intranasally immunized with H5N1 VLPs containing 0.3 µg HA. Significant levels of H5 virus specific IgG antibodies were induced in nasal and tracheal washes as well as in lung extracts of mice after prime-boost immunization with H5N1 VLPs (H5VLP group in Fig. 8A). H5 virus-specific IgA antibodies were also induced in lung extracts (Fig. 8B). To determine the anamnestic virus-specific mucosal antibody responses, VLP-immunized and PBS control mice were re-stimulated with inactivated rgΔH5N1 virus (5 µg) *in vivo* via an intranasal route. Respiratory secretions were collected 6 days later and analyzed to determine the concentration of mucosal antibodies to H5N1 by ELISA. H5 virus-specific IgG and IgA antibodies were induced faster and accumulated to higher levels at mucosal sites of previously VLP immunized mice (H5VLP+H5i) as compared to the PBS control mice (PBS+H5i). These results suggest that intranasal immunization with H5N1 VLPs can induce a memory response that mediates rapid virus-specific mucosal IgG and IgA responses upon subsequent virus antigen exposure.

### Anamnestic responses of cytokine producing cells

**Figure 9:**
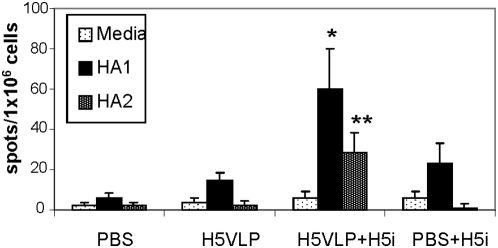
IFN-γ ELISPOT. Naïve, unimmunized mouse control, H5VLP, H5VLP+H5i and N (naïve)+H5i, groups of mice (n = 6 for each group) intranasally immunized as described in the legend of Fig. 8. Spleen cells were collected from individual mouse and stimulated with H5 HA1 or H5 HA2 peptide pool derived from A/Thailand/16/04 (H5N1). * and ** indicate significant differences between H5VLP+H5i and H5VLP or PBS+H5i group (p<0.05 and p<0.005, respectively) as determined by Student's 2-tailed t-test.

To examine specific T cell memory induced by H5N1 VLPs, spleen cells were harvested at 4 weeks post prime-boost immunization and analyzed for their IFN-γ secreting splenocytes upon the stimulation with an H5 HA specific peptide pool derived from A/Thailand/16/04 (accession number APB51982, H5N1) which has over 99% homology to VN/04 HA (Fig. 9). Higher numbers of IFN-γ secreting splenocytes specific to the HA peptide pool were observed in the immunized animals as compared to control mice. To determine the recall responses of memory T cells *in vivo*, splenocytes were analyzed for IFN-γ producing cells at day 6 after intranasal stimulation with inactivated rgΔH5N1 virus. H5N1 VLP-immunized mice showed significantly higher numbers of IFN-γ secreting cells upon stimulation with H5 HA peptide pools corresponding to both HA1 and HA2 subunits of H5 HA as compared to those of H5-naive mice at the time of viral antigen challenge with inactivated rgΔH5N1 virus. Therefore, H5N1 VLP immunization of mice elicits IFN-γ cytokine secreting T cells specific to both HA1 and HA2 peptides, which can be recalled to respond to virus exposure with fast kinetics.

## Discussion

Our results demonstrate that intranasal immunization of mice with H5N1 VLPs in the absence of adjuvant induced long-lasting protective immunity, providing effective protection from disease and death following a high dose lethal challenge with human isolate of highly pathogenic avian influenza virus (H5N1). Also, mice immunized with H5N1 VLPs elicited virus specific plasma and memory B cells as well as mucosal antibodies and IFN-γ secreting cells, which undergo rapid *in vivo* expansion upon re-encounter with viral antigen. These results highlight the potential of non-replicating particulate VLPs as a promising subunit vaccine whose manufacture does not require fertile eggs.

Inactivated detergent split or subunit H5N1 vaccine produced in eggs given without adjuvants have revealed poor immunogenicity in preclinical and clinical studies, requiring a high dose or two dose immunization protocol. In humans, high doses of baculovirus-expressed H5 HA or inactivated subunit vaccines produced in eggs (two 90 µg HA doses) were needed to induce antibody responses that were expected to be protective in 54 to 58% of individuals vaccinated [23], [39]. Two doses of adjuvanted inactivated H5N1 whole virus or split vaccine containing 3 µg HA were used to induce protective immunity or to improve protection efficacy in mice [26], [40], [41]. A single dose of 10^6^ pfu of live attenuated vaccine virus was weakly immunogenic and could protect mice from lethality despite high challenge virus replication in the respiratory tract, whereas two doses of live vaccine were required to protect mice and ferrets from pulmonary replication of challenge H5N1 viruses [17]. A recent study demonstrated that a single dose of H5N1 (A/Indonesia/05/05) VLPs containing 3 µg HA delivered via intramuscular immunization provided protection against challenge with homologous reassortant H5N1 virus (10 LD_50_) in mice [31]. Our study showed that two doses of H5N1 VLPs (VN/04) containing 0.3 µg HA delivered intranasally provided complete protection from lethal challenge with wild-type VN/04 virus in mice without loss in body weight. Protection from lethal challenge with VN/04 virus was also observed with doses containing as low as 0.1 µg H5 HA in VLPs despite a transient body weight loss. Taken together, these results indicate that influenza H5N1 VLPs are highly immunogenic in the preclinical mouse model. These studies point towards the need to evaluate intranasal VLP H5N1 vaccine in humans to determine its ability to induce protective immune responses comparable to those of the conventional egg-grown influenza subunit vaccines with adjuvant.

Immune correlates of protection against H5N1 viruses have not been well defined either in animals or in humans. Hemagglutination inhibition (HAI) is a widely used serological assay for measuring functional influenza-specific serum antibodies to HA following immunization with inactivated vaccines. However, this assay may be less predictive for avian H5N1 viruses whose pathogenesis differs from that of seasonal influenza viruses. Indeed, previous studies found less correlation between HAI titer and protection against H5N1 virus infection [17], [31]. Mice immunized with a single intramuscular dose of H5N1 VLPs, or recombinant subunit H5 HA (A/Indonesia/05/05) or intranasal live attenuated reassortant virus responded with low or no detectable HAI titers, but survived a lethal challenge [17], [31]. Similarly, in this study, low or negligible HAI titers and neutralizing activities were detected in the sera of mice that were 100% protected from lethal challenge infections (100 MLD_50_) by wild type VN/04 H5N1 virus. Our study and a previous report [31] indicate that induction of IFN-γ secreting T cell responses may correlate with reduced viral replication in lungs and host protective immunity. In a comparative study, H5N1 (A/Indonesia/05/05) VLP vaccine was over 100-fold more effective in lowering lung viral titers and morbidity than vaccination with the equivalent amount of subunit H5 HA immunogen similar to the egg-derived split vaccine [31]. The IgG2a isotype predominance in the H5-specific humoral antibody response indicates that intranasal immunization with H5N1 VLPs induced Th1 biased immune responses. In support of the postulated Th1 type response, intranasal immunization with H5N1 VLPs induced formation of memory T cells that could readily secrete IFN-γ upon HA peptide stimulation particularly by the HA2 region peptide. These memory cells could be rapidly recruited to the respiratory tract and might contribute to lowering lung viral titers. Previous studies have shown that T cell responses may play a role in providing protection. Intramuscular immunization with H5N1 VLPs [31] and vaccination with HA-expressing adenoviruses [24] suggested that T cell responses to the HA2 region contributed to protection against heterologous homotypic strains in the absence of a strong humoral neutralizing response.

Inclusion of additional influenza proteins besides HA may partially contribute to protection particularly when neutralizing (and/or HAI) antibody responses are low or undetectable. The NA component of H5N1 VLPs may contribute to protection against morbidity and mortality in the absence of neutralizing activities as reported previously [42]–[46]. Similarly, the M1 component of H5N1 VLPs elicits CD8 T cell responses that contribute to partial protection as noted earlier [47], [48]. Studies on the possible role of these proteins in VLP-induced protective immunity are under way.

A primary goal of vaccination is to induce memory responses that will provide long-lived protection against severe disease by the pathogen. However, currently available inactivated influenza vaccines do not induce long-lasting immune responses. Antibody levels elicited by inactivated influenza virus vaccine administered intramuscularly have been observed to gradually decline by 75% over an eight-month period [49]. We have shown that intramuscular immunization of mice with influenza VLPs containing H1 HA (PR/8/34) induced long-term protective immunity over 14 months [30]. In this study, intranasal immunization of mice with H5N1 VLPs induced virus specific antibodies that lasted for the 7 month period analyzed. Furthermore, immunized mice challenged with a high dose of wild type virus were completely protected from morbidity and mortality. Also, virus specific antibody secreting plasma cells and memory B cells widely regarded as long-lived were identified in bone marrow and spleens, respectively. These results are consistent with the postulated generation of host-protective memory immune responses by H5N1 VLP immunization.

VLPs administered intranasally to BALB/c mice are likely to be taken up by microfold epithelial cells in the nasal compartment and transferred to nasal associated lymphoid tissue to subsequently traffic through peripheral lymph nodes into spleen [50], inducing mucosal as well as systemic immune responses. The generation of memory immune responses was further confirmed by assays using both *in vivo* antigen challenge and *in vitro* stimulation. In addition to long-lived plasma cells maintaining serum antibody levels, intranasal VLP immunization induced memory B cells that rapidly expand and differentiate into antibody secreting cells after exposure to viral antigen. Detection of antibody secreting cells in bone marrow suggested that a population of antibody secreting cells derived from memory B cells may traffic to the bone marrow. Also, VLP-immunized mice responded to viral antigen in vivo with virus-specific anamnestic mucosal IgG and IgA antibody kinetics, whereas in vitro stimulation revealed influenza-specific IFN-γ secreting splenocytes. To our knowledge, this is the first reported evidence of induction of memory B and T cell responses by intranasal immunization with H5N1 VLPs, and their rapid expansion in both mucosal and systemic sites upon viral antigen encounter.

Adult populations have a certain level of pre-existing immunity against seasonal influenza virus, but most humans still respond to annual vaccination. Therefore, it is likely that host immune responses to VLP antigens would not be affected by the presence of pre-existing immunity to influenza A. Nonetheless, it will be important to determine the immunogenicity of pandemic H5 VLPs in the presence of immunity to seasonal influenza virus. Also, immune responses to VLPs in aged mice will provide helpful insight into vaccinating the elderly populations which are more susceptible to influenza infection. An influenza vaccine providing broadly cross protective immunity is highly desirable. Mice immunized intranasally or intramuscularly with A/PR/8 (H1N1) VLPs were found to be protected against heterologous challenge with A/WSN (H1N1) virus [29], [30]. In another recent study, mice vaccinated with A/Indonesia/05 VLPs were reported to be protected against lethality after heterologous challenge with an A/Viet Nam H5N1 reassortant virus [31]. Thus, mice intranasally immunized with H5N1 (A/VN) VLPs would be expected to protect against heterologous H5N1 strains such as A/Thailand/16/04 or A/Indonesia/05/05 although the extent of cross protection among different influenza strains remains to be determined.

In summary, unadjuvanted influenza H5N1 VLPs are immunogenic in mice and were found to induce long-term protective immunity as well as to generate immunologic memory for rapid recall responses. Non-replicating particle-based VLP approaches circumvent the need for handling live influenza viruses in vaccine manufacturing. We have not observed any abnormal symptoms in mice immunized with pandemic influenza H5N1 VLPs or seasonal influenza VLPs. Therefore, the results of this preclinical study indicate that influenza H5N1 (VN/04) VLPs are highly immunogenic and have the potential to be developed as an effective vaccine inducing long-term protective immunity.

## Materials and Methods

### Virus and cells

The highly pathogenic avian influenza H5N1 virus A/Viet Nam/1203/04 (VN/04) isolated from a fatal human infection and the reassortant virus (rgΔH5N1) produced by reverse genetic techniques [17], [26], [27] were used in the study. The rgΔH5N1 virus contains HA modified at the cleavage site (Fig. 1A) and NA derived from H5N1 virus VN/04 and internal proteins from H1N1 virus A/Puerto Rico/8/34 (PR/8/34). A/Puerto Rico/8/34 (H1N1) was obtained from American Type Culture Collection (ATCC). Viruses were propagated in the allantoic cavity of 10-days-old embryonated chicken eggs. Virus containing allantoic fluid was harvested, aliquoted and frozen at −80°C until used in experiments. Purified virus was prepared by equilibrium and differential sucrose density gradient separations [51]. VN/04 virus was used in animal challenge experiments and for the isolation of viral RNA. All experiments with live H5N1 virus were performed in biosafety level 3 enhanced laboratory facilities. Reassortant virus rgΔH5N1 was inactivated with formalin, concentrated and purified by centrifugation, and used as an ELISA antigen as well as in vivo and *in vitro* viral antigen stimulator. Spodoptera frugiperda Sf9 cells were maintained in suspension in serum free SF900II medium (GIBCO-BRL) at 27°C in spinner flasks at a speed of 70–80 rpm. Madin-Darby canine kidney (MDCK) cells were obtained from ATCC and were cultured in Dulbecco's Modified Eagle's Medium supplemented with 10% fetal bovine serum.

### DNA constructs, recombinant baculoviruses, and production of VLPs

The cDNA fragments encoding HA, NA, or M1 proteins derived from influenza H5N1 virus (VN/04) were generated using RT-PCR and cloned into the pCI plasmid vector (Promega). The H5 HA cDNA was mutated to delete the polybasic amino acids in the HA1/HA2 cleavage region as shown in the Fig. 1A. The HA encoding gene in the pCI vector was treated with Xho I, Klenow polymerase fragment, then subsequently digested with Sal I, and cloned into the pFastBac plasmid, a baculovirus (BV) transfer vector (Invitrogen). The NA cDNA in pCI was PCR-amplified using the following primers: F-NA-EcoRI, 5′ AAGAATTC CCACCATG AAT CCA AAT CAG AAG ATA ATA 3′ and R-NA-XbaI, 5′ AG TCTAGA CTA CTT GTC AAT GGT GAA TGG 3′. The M1 cDNA was PCR-amplified using the primers: F-M1-Sma I, 5′ TCC CCCGGG CCACCATGAGCCTT CTG ACC GAG GTC 3′ and R-M1-Xba I, 5′-TTACT TCTAGA TTACTTGAATCG CTG CAT CTG 3′ (underline denotes restriction enzyme recognition sites). The PCR-amplified NA and M1 encoding DNA fragments were digested with corresponding restriction enzymes and cloned into the pFastBac plasmid under the polyhedron promoter, and confirmed by DNA sequencing. For the generation of recombinant BVs (rBVs) expressing HA, NA, M1 respectively, recombinant Bacmid baculovirus DNAs (rAcNPV) were isolated from transformed DH10Bac cells with pFastBac plasmid constructs and used to transfect Spodoptera frugiperda Sf9 insect cells following the manufacturer's instructions (Invitrogen). The virus titer was determined with a Fast Plax titration kit according to the manufacturer's instructions (Novagen, Madison, WI). The expression of H5N1 structural proteins in infected insect cells was confirmed by western blot using rabbit anti-HA or NA polyclonal antibodies (ProSci Inc.,) or mouse anti-M1 antibody (Serotec).

To produce influenza H5N1 VLPs, Sf9 insect cells were co-infected with rBVs expressing HA, NA, and M1, and VLPs were harvested from the culture supernatants by centrifugation and purified using sucrose gradient ultracentrifugation as described [29], [30]. HA contents in VLPs were determined by western blot in comparison with the purified H5 HA protein (Biodefense and Emerging Infections Research Resources Repository) derived from the influenza virus VN/04 as a standard. The electron microscopic examination of purified H5N1 VLPs was carried out as described previously [29]. The functional characterization of HA on VLPs was performed by analyzing hemagglutination activity and trypsin cleavability assays as described [29], [30]. Influenza H5N1 VLPs used in this study contained approximately 10% H5 HA relative to total protein of VLPs, similar to previously reported values for H1N1 VLPs [30].

### Immunization and challenge

Female inbred BALB/c mice (Charles River) aged 6 to 8 weeks were housed in the facility of Emory University following the approved IACUC protocol. Groups of mice (6 mice per group) were intranasally immunized with 50 µl of influenza H5N1 VLPs containing 0.1 µg or 0.3 µg of H5 HA at weeks 0 and 4 under a slight anesthesia condition with isofluorane. Two groups of 6 mice each were mock-immunized with PBS or immunized with 3 ug of inactivated A/PR/8/34 purified following the same schedule as for VLP groups. H5N1 VLP-immunized and control mice were transferred to the enhanced animal biosafety level 3 facilities in the Centers for Disease Control and Prevention (Atlanta, GA, USA) for virus challenge studies. Animals were anesthetized by isoflurane (Butler Animal Health Supply) inhalation and inoculated intranasally with 50 µl of sterile PBS containing 10^3^ plaque forming units (pfu), approximately 100 of 50% mouse lethal doses (MLD_50_) of VN/04 virus. Mice were observed and weighed daily starting immediately before challenge, to monitor health status. The humane endpoint of the challenge studies was body weight loss of ≥20% relative to the weight at the time of challenge inoculation. Animal study protocols were performed in compliance with institutional and federal guidelines. For determination of lung viral titers, mucosal immune responses, and cytokine ELISPOT and memory B cell assays, additional sets of mice were intranasally immunized with H5N1 VLPs containing 0.3 µg H5 HA, and some groups of these mouse groups were intranasally administered with 5 µg of inactivated reassortant rgΔH5N1 virus 6 days earlier prior to euthanasia and organ sample collection.

### Antibody responses, isotypes, and hemagglutination inhibition titer (HAI)

Blood samples were collected by retro-orbital plexus puncture before immunization and at various time points post-immunization (at week 2 after priming and at week 6 and 28 after boosting). Influenza virus specific antibodies IgG, isotypes IgG1, IgG2a, IgG2b, and IgA were determined in sera by enzyme-linked immunosorbent assay (ELISA) as described previously [29]. 96-well microtiter plates (Nunc Life Technologies, Rochester, NY.) were coated with 0.4 µg of sucrose gradient purified egg-grown inactivated rgΔH5N1 virus in 100 µl of coating buffer (0.1 M sodium carbonate, pH 9.5, 4 µg inactivated rgΔH5N1 virus per ml) at 4°C overnight. For antibody titers, dilutions giving 2-fold higher OD_450_ values than the standard deviation compared to the those of naïve control samples were considered as positive. Hemagglutination-inhibition (HAI) titers were determined by standard methods using 4 HA units of inactivated rgΔH5N1 and 1-% horse erythrocyte suspension [52]. Microneutralization assays were performed as described previously [53], [54].

### Plasma cells and memory responses

A group of mice (n = 6) was intranasally immunized with influenza H5N1 VLPs containing 0.3 µg HA at weeks 0 and 4. Cells from bone marrow and spleens of mice at week 4 post immunization or control mice (PBS) were prepared as described [55]. Freshly isolated bone marrow and spleen cells (1×10^6^ cells) were added to the Multiscreen 96-well filtration plates (Millipore) coated with inactivated rgΔH5N1 virus antigen. After incubation for 18 or 72 hrs at 37°C with 5% CO_2_, bound antibody was detected using an ELISPOT kit with HRP-conjugated anti-mouse Ig and diaminobenzidine for color development following the manufacturer's instructions (Research Genetics). The antibody-secreting cell spots were recorded per 10^6^ input cell numbers. In another set of experiments to determine anamnestic responses, H5N1 VLP-immunized mice were administered with 5 µg of inactivated rgΔH5N1 virus intranasally 6 days earlier, and bone marrow and spleen cells were analyzed for cytokine production and virus-specific antibody secreting cells.

### Cytokine assays

Antibodies against mouse cytokines used in cytokine ELISPOT assays were purchased from BD/Pharmingen (San Diego, Calif.). Anti-mouse interferon-γ (IFN-γ) and IL-4 antibodies (3 µg/ml in coating buffer) were used to coat Multiscreen 96-well filtration plates (Millipore). Freshly isolated splenocytes (1.5×10^6^ cells) were added to each well and stimulated with an HA1 or HA2 subunit peptide pool at a concentration of 10 µg/ml. Peptide pools are 15-mer peptides with 11 amino acid overlap derived from A/Thailand/16/04 (H5N1) which has over 99% homology to VN04 HA. The plates were incubated for 36 hrs at 37°C with 5% CO_2_. Development and counting of cytokine ELISPOTs were performed as described [29].

### Viral titers

Mice in groups of 4 received intranasally as described above two doses of 0.3 µg HA each of H5N1 VLPs. Control group of 4 animals received PBS alone. Four weeks after the second VLPs immunization mice were inoculated with 10^3^ pfu (100 MLD_50_) of VN/04 virus as described above. Mice were euthanized on day 4 after virus inoculation, and lungs, brain, and spleen were harvested. Organs were homogenized in 1 ml of sterile PBS with antibiotics; homogenates were clarified by centrifugation. Virus titers were determined by plaque assay in MDCK cells and expressed as log_10_ of pfu in 1 ml of organ homogenate. The limit of virus detection was 50 pfu (1.69 log_10_) per 1 ml.

### Statistical analysis

All immunological parameters were recorded for individuals within groups. Statistical comparisons of data between groups were calculated by Student's 2-tailed t-test. A value of P<0.05 was considered significant
